# Long-Term Treatment with Romidepsin in Patients with Peripheral T-Cell Lymphoma

**DOI:** 10.1155/2016/8175957

**Published:** 2016-08-25

**Authors:** Claudius Irlé, Jonathan Weintraub

**Affiliations:** ^1^Hôpital de la Tour Unite d'Oncologie-Hematologie, Meyrin, Switzerland; ^2^Viollier Weintraub, Geneva, Switzerland

## Abstract

Peripheral T-cell lymphomas (PTCLs) are a heterogeneous group of aggressive non-Hodgkin lymphomas. Angioimmunoblastic T-cell lymphoma (AITL) is a common subtype of PTCL, and patients with AITL typically have a poor prognosis with limited treatment options. Clinical studies have demonstrated the activity of romidepsin, a structurally unique, potent, bicyclic class 1 selective histone deacetylase inhibitor, in patients with relapsed or refractory AITL. In the case presented herein, we describe a patient treated with single-agent romidepsin at first diagnosis of AITL, resulting in complete remission for over 2 years and leading to the use of maintenance dosing. The patient eventually underwent a successful autologous stem cell transplant. This case illustrates the successful use of romidepsin for the long-term treatment of a patient with AITL in a clinical setting. Maintenance dosing may be an option for patients who have an extended response to romidepsin in order to optimize outcomes and to prolong time to the next subsequent line of therapy. In our case, the patient was able to remain in complete remission for more than 1 year while receiving maintenance dosing of romidepsin.

## 1. Introduction 

Peripheral T-cell lymphomas (PTCLs) are a diverse group of aggressive T-cell and natural killer-cell malignancies associated with poor prognosis [1, 2]. Patients with all subtypes of PTCL are most often treated with anthracycline-based chemotherapy regimens in the first line, but most responders experience rapid relapse [1–5]. The epigenetic modifying agent romidepsin is a structurally unique, potent, bicyclic class I selective histone deacetylase inhibitor [6–8] approved by the US Food and Drug Administration for the treatment of patients with PTCL who had received ≥1 prior therapy and patients with cutaneous T-cell lymphoma (CTCL) who had received ≥1 prior systemic therapy [9]. In the pivotal phase 2 study in PTCL, romidepsin (14 mg/m^2^ on days 1, 8, and 15 of 28-day cycles) demonstrated a 25% objective response rate, including 15% of patients with confirmed/unconfirmed complete response (CR) [10, 11]. Several patients experienced durable responses, with a median duration of response of 28 months [11]; the longest response is ongoing at 56 months in a patient with angioimmunoblastic T-cell lymphoma (AITL) [12]. As a result, the trial protocol was amended to allow (but not mandate) patients treated for ≥12 cycles to receive maintenance dosing of 2 rather than 3 doses per 28-day cycle [11]. Patients treated for ≥24 cycles who had received 2 doses per cycle for ≥6 cycles could be treated at maintenance dosing of 1 dose per cycle [11]. There are no current guidelines in place for prolonged use of romidepsin in a clinical setting, although an observational study to examine the long-term use of romidepsin in patients with CTCL is under way (NCT02296398).

## 2. Case Presentation

We report the case of a 54-year-old woman who presented in September 2010, 3 weeks following a dental extraction due to a tooth abscess with fever, at the Geneva University Hospital emergency unit with joint pain, fatigue, loss of appetite, nausea, and fever, with multiple sensitive bilateral cervical lymphadenopathy. Written informed consent was obtained from the patient for publication of this case report. At the time of presentation, she was in good general health without fever or jaundice, and both cardiopulmonary and abdominal examinations were unremarkable. She had a mild micronodular skin rash on her ankles. Clinical examination revealed the presence of 1.5 cm cervical nodes and smaller axillary nodes. A complete blood count showed 10.8 g/L white blood cells (9% eosinophils and 1% plasma cells) and 282 g/L platelets. Her electrolytes and liver and renal function tests were normal, and serologies for cytomegalovirus, Epstein–Barr virus (EBV),* Borrelia*, and human immunodeficiency virus were all negative. A chest X-ray showed suspect perihilar areas compatible with lymph node enlargements. She was discharged from the emergency unit to her general practitioner with a diagnosis of inflammatory syndrome following her dental extraction and treated with ibuprofen and paracetamol. On 11 September 2010, it was recommended that she undergo further evaluation for hematologic and inflammatory conditions (e.g., sarcoidosis).

A computed tomography (CT) scan was performed on 16 September 2010, which showed multiple homogeneous lymphadenopathies in all areas examined, with no hepatosplenomegaly. The patient appeared to have a lymphoproliferative condition that was clinically stable. In December 2010, she was referred to Dr. Irle at the Centre D'Onco-Hématologie de Meyrin. A positron emission tomography (PET)/CT scan was performed on 8 December 2010, which showed numerous lymphadenopathies extending from the cervical areas to supraclavicular and axillary nodes as well as mediastinal, retroperitoneal para-aortic areas extending to as far as the inguinal nodes. Additionally, there were mesenteric lymph nodes. A core biopsy was performed on 9 December 2010, and an enlarged inguinal node and pathology were indicative of an atypical lymphoid proliferation suspicious for non-Hodgkin lymphoma; this diagnosis was confirmed by an external expert pathologist on 30 December 2010. On 3 February 2011, a CT scan showed no change from the previous scan. On 22 February 2011, the patient had a surgical excision biopsy of an inguinal lymph node, leading to a diagnosis of mixed-cellularity EBV+ classical Hodgkin disease, both locally and externally. A bone marrow examination on 3 March 2011 showed no evidence of disease infiltration in the bone marrow.

On 9 March 2011, the patient began treatment with standard-dose AVBD (doxorubicin 25 mg/m^2^, bleomycin 10 U/m^2^, vinblastine 6 mg/m^2^, and dacarbazine 375 mg/m^2^) and prednisone 50 mg/m^2^ every 14 days in 28-day cycles. On 27 April 2011, following 2 cycles of treatment, a PET/CT scan showed a complete metabolic response. On 23 August 2011, following 8 cycles of treatment, a CT scan showed near-complete regression of the lymph nodes. However, some slightly enlarged cervical nodes measuring 12 mm were found (submaxillary, internal jugular). A PET scan was also performed on 17 September 2011, which showed slight enhancement of the metabolic uptake in the lateral cervical, axillary, and inguinal nodes (standardized uptake values (SUVs), 1.3–1.5) compared with the scan from April 2011.

On 22 September 2011, a biopsy of a cervical lymph node was performed; pathology indicated reactive lymphadenitis with cortical hyperplasia, without evidence of residual lymphoma. A second opinion on pathology was then requested; overall immunohistochemical and molecular analysis on all available biopsy specimens from 31 October 2011, 1 November 2011, and 9 November 2011 was performed. From this analysis, it was concluded that the most likely diagnosis was AITL type 3 according to Attygalle, with the same monoclonal T-cell receptor (TCR) rearrangement found in each biopsy specimen.

On 16 January 2012, the patient began treatment with romidepsin 20 mg (14 mg/m^2^) on days 1, 8, and 15 of 28-day cycles. While romidepsin side effects included rapid and persistent ageusia and substantial fatigue, no hematologic toxicities were reported. On 9 March 2012, a PET/CT scan was normalized metabolically and showed regression in the suspicious lymph nodes to normal size; it was concluded that the patient was in morphological and metabolic CR, and a CT scan on 5 June 2012 showed no change. Through the first 12 months of treatment, the patient continued with dosing on days 1, 8, and 15 of 28-day cycles at the same dose. A PET/CT scan on 17 September 2012 showed small residual cervical nodes that were nonsuspicious but had low-grade metabolic activity (SUV, 1.4 versus 0.9). Starting 15 January 2013, the patient initiated maintenance dosing and received romidepsin on days 1 and 15 of 28-day cycles. She continued to be evaluated by routine trimestral PET/CT scans and remained in CR without evidence of metabolic or morphological recurrence. On 24 November 2014, relapse was diagnosed by PET scan and confirmed by biopsy on 2 December 2014. While the histology was inconclusive, the molecular analysis of TCR showed a strong clonal signal confirming the relapse. Starting on 5 January 2015, romidepsin was introduced on days 1, 8, and 15 of 28-day cycles and was given for 3 months. A PET/CT scan on 17 March 2015 showed continuing activity, with relapsed nodes. At that point, treatment with romidepsin was stopped and chemotherapy with dexamethasone, cyclophosphamide, and oxaliplatin (DAOx) was given. After 4 treatment cycles, a PET/CT scan showed that the patient was in morphological and metabolic CR. Therefore, we proceeded in August 2015 with stem cell harvest using granulocyte colony-stimulating factor (G-CSF) stimulation to mobilize peripheral blood stem cells (PBSCs), which were harvested without difficulty in satisfactory numbers. An autologous stem cell transplant was then performed successfully in September 2015, with rapid and full engraftment. A posttransplant CT scan was performed 1 month after transplant and showed no abnormality.

## 3. Discussion 

The initial diagnosis of classical Hodgkin lymphoma was made following lymph node biopsy and was based on histology and EBV positivity, although early suspicion for AITL was noted. A diagnosis of AITL was made after additional biopsy and analyses of the available immunohistochemical and molecular data in consultation with an external expert. In our opinion, based on post hoc analysis of all the data, the patient had AITL from the beginning. PCR analyses of material from the original core biopsy and from biopsies after progression and after relapse were positive for a TCR clone.

Nevertheless, use of romidepsin as the first treatment upon diagnosis of AITL resulted in a CR of over 2 years and led to the use of maintenance dosing in a clinical setting. In a recent subanalysis of patients with AITL from the romidepsin pivotal study, all 5 patients with CR ≥ 12 months received maintenance dosing [12]. To optimize patient outcomes and prolong the time to the next subsequent therapy, guidelines for prolonged romidepsin treatment are needed. Until they are available, investigators may use the pivotal study protocol amendment as a point of reference. Not only did long-term romidepsin allow the patient to obtain a prolonged maintained remission, but it also did not compromise the successful rescue treatment, with an uncomplicated harvest of G-CSF-mobilized PBSCs, once a second remission was obtained with DAOx chemotherapy. Following the conditioning regimen and reinfusion of the rescue PBSCs, engraftment was rapid and resulted in a complete peripheral hematologic recovery.
